# Research on the Surface Evolution of Single Crystal Silicon Mirror Contaminated by Metallic Elements during Elastic Jet Polishing Techniques

**DOI:** 10.3390/ma12071077

**Published:** 2019-04-02

**Authors:** Wanli Zhang, Feng Shi, Yifan Dai, Yaoyu Zhong, Ci Song, Ye Tian

**Affiliations:** College of Artificial Intelligence and Automation, National University of Defense Technology, 109 Deya Road, Changsha 410073, Hunan, China; zhangwanli17@nudt.edu.cn (W.Z.); dyf@nudt.edu.cn (Y.D.); zhongdayuu@gmail.com (Y.Z.); sunicris@163.com (C.S.); tianyecomeon@sina.cn (Y.T.)

**Keywords:** single crystal silicon, metallic contaminant, EJP, SAE, surface quality, infrared laser system

## Abstract

Metallic elements can contaminate single crystal silicon mirror during ion beam etching (IBE) and other postprocessing methods, which can affect the performance of components in an infrared laser system. In this work, scanning electron microscope (SEM) and atomic force microscope (AFM) were used to characterize the distribution of contaminant represented by aluminum (Al). After characterizing contaminated area, elastic jet polishing (EJP), EJP, and static alkaline etching (SAE) combined technique were used to process the mirror. The morphology and laser-induced absorption were measured. Results show that metallic elements can mix with silicon and generate bulges due to the sputtering effect. In addition, SAE and EJP combined technique can remove metallic contaminant and stabilize the surface quality. Research results can be a reference on conducting postprocessing technologies to improve laser damage resistance property of single crystal silicon mirror in infrared laser system.

## 1. Introduction

**Figure 1:**
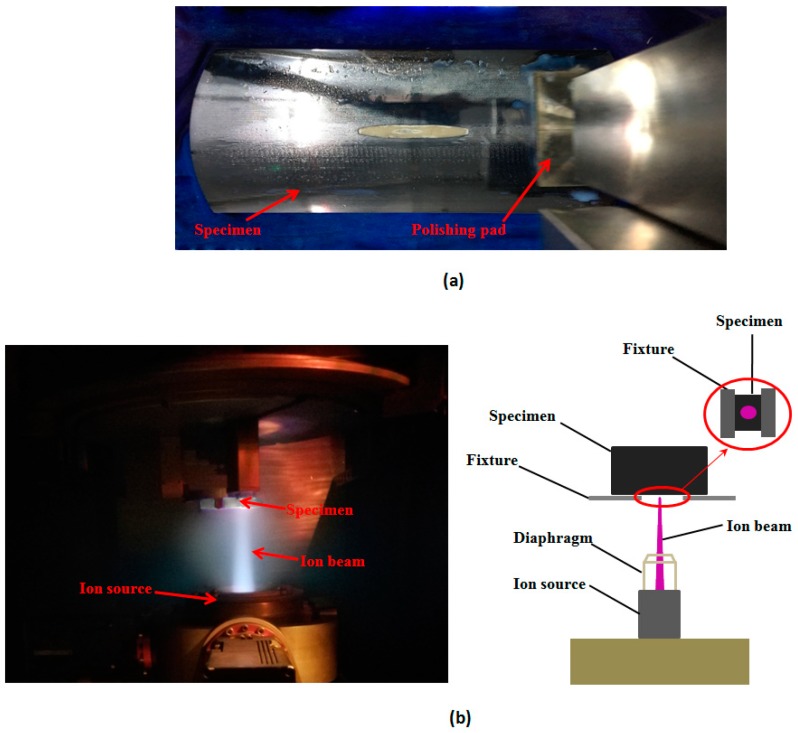
Postprocessing of single crystal silicon mirror: (**a**) computer-controlled optical surfacing (CCOS) processing and (**b**) ion beam etching (IBE) processing.

Single crystal silicon is of critical importance in the development of high-power laser systems [1,2]. Improving the high-precision and low-defect performance of single crystal silicon mirror is one of the basic pursuing objectives for high-power laser systems [3,4]. Advanced techniques such as computer-controlled optical surfacing (CCOS) and IBE, shown in Figure 1, are used as postprocessing techniques of single crystal silicon mirror [5,6]. In CCOS processing, chemical action coexists with mechanical action, which improves the efficiency. For IBE processing, materials can be removed determinately and mechanical defects would not be introduced because of its noncontact processing mode [7]. Tian used immersed CCOS and IBE combined technique to process single crystal silicon cylindrical mirror. After completion, Peak-to-valley (PV) value of the mirror was less than λ/4 and the roughness root mean square (RMS) value was below 1 nm [7]. However, these two methods have shortcomings. For CCOS processing, the planar surface can be polished with high precision. As for complex surface including aspheric surface, the polishing lap is not fitted with an optical surface, seriously influencing machining accuracy, which may introduce mechanical defects [5]. The metallic materials of polishing lap can also contaminate the component. For IBE processing, it can remove mechanical defects without causing damage. However the residual metallic contaminant introduced in CCOS processing still remains on the surface. In the meantime, metallic materials of ion source or diaphragm also can be brought into the surface owing to the action of the ion beam, which has an influence on surface quality [8,9,10]. As a kind of defect, the metallic contaminant can cause extra absorption, which may lead to laser-induced damage [11,12,13,14]. Sun found that external metallic contaminant limited the laser-induced damage threshold (LIDT) of fused silica. After chemical etching, LIDT increased from 4.7 J/cm^2^ to 7.3 J/cm^2^ [15,16]. Tian considered that precursors including metallic contaminant could cause extra laser-induced absorption [11].

In the actual fabrication process, with continued advances in single crystal silicon mirror, the required specification for ultraclean wafer surfaces becomes very challenging [17]. During traditional postprocessing techniques, it is difficult to remove the metallic contaminant. In order to process the single crystal silicon without causing secondary pollution, new flexible postprocessing techniques are needed [18]. At present, fluid polishing methods such as jet polishing are used as postprocessing techniques, which can achieve high-precision, pollution-free, low-defect fabrication. Song used jet polishing to polish fused silica. After jet polishing, the roughness of specimen was less than 0.7 nm and the content of contaminant significantly decreased [19].

**Figure 2:**
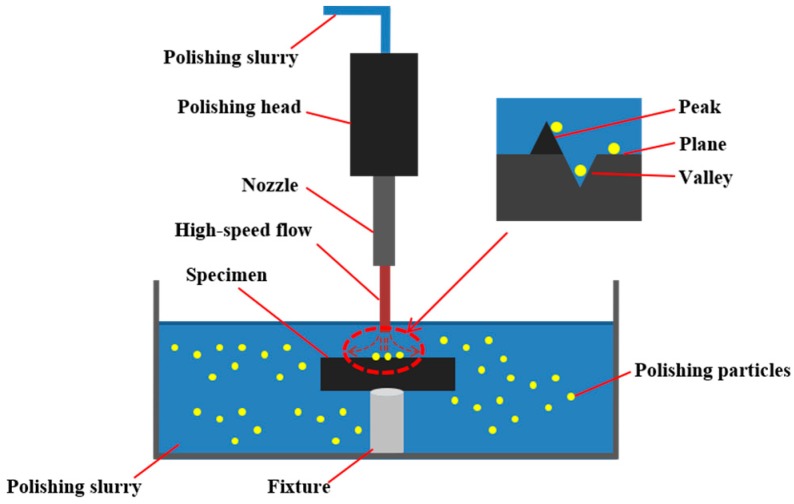
Mechanism of jet polishing.

Fundamentally, the mechanism of action of different jet polishing methods is the same, as shown in Figure 2. During jet polishing, a high-speed flow crashes onto the single crystal silicon, which is immersed in the polishing slurry, to remove materials. It should be noted that the whole process contains mechanical action and chemical action. When immersed in polishing slurry, polishing particles collide with the surface and contact it through adsorption. Then, combinations are removed through the shearing force provided by the high-speed flow. For low-defect fabrication, EJP processing could remove the materials without causing secondary damage. To achieve processing in the elastic region, the key to EJP is to control the particle size and velocity of polishing flow. In EJP processing, materials of the surface at the “peak” are absorbed by polishing particles first, then the “plane”, and last is the “valley” according to quantum chemical simulation [20,21], so as the sequence of material removal. The mechanism determines that the surface tends to be smooth during EJP processing. Generally, SiO_2_ and TiO_2_ are used as polishing particles. To avoid introducing other elements, SiO_2_ was selected in the process of single crystal silicon mirror. It is noteworthy that chemical etching is used before jet polishing in order to expose subsurface damage [19]. For single crystal silicon, a KOH solution is frequently used as the etchant [22]. Studies proved that a chemical etching and jet polishing combined technique can be used as a postprocessing technique for fused silica [19,23,24]. As for single crystal silicon, studies on its evolution needs to be explored [20,25]. 

The purpose of this work is to research the evolution of optical surface contaminated by metallic elements for the EJP technique or SAE and EJP combined technique, respectively. The article is organized as follows. Section 1 is the introduction. The materials and methods are shown in Section 2. Testing and results of surface evolution are described in Section 3. Section 4 is the discussion about problems arising in the experiments and Section 5 is conclusion. Above all, experimental results can be a reference on using the SAE and EJP combined technique to improve the performance of single crystal silicon in infrared system. The paper also represents a preliminary but potential study, which can be adopted in the postprocessing of single crystal silicon mirror.

## 2. Materials and Methods 

### 2.1. Materials and Preprocessing 

Two single crystal silicon substrates named sample #1 and sample #2 (orientation: 100, size: Φ 50 mm × 5 mm, shape: plane, roughness Rq value: 0.650 nm) were prepared by a vendor called YUCAI Optical technology, LTD. (Tianjin, China). The substrates are polished by chemical/mechanical polishing (CMP). Then, two samples are processed by the IBE technique (instrument: KDIBF650L-T, National University of Defense Technology, Changsha, Hunan, China). During the IBE process, metallic elements could contaminate single crystal silicon mirror. Aluminum was selected as the analysis object because of the application of aluminum alloy components and its relatively high content. Parameters of IBE are listed in Table 1.

### 2.2. Methods

#### 2.2.1. Postprocessing 

Sample #1 was used to study the surface evolution during EJP. Firstly, we used EJP to remove 2 nm, 4 nm, and 6 nm depths of sample #1 in turn; the morphology of bulges will be investigated. Secondly, removal depths of 20 nm, 40 nm, 60 nm, 80 nm, and 100 nm were removed during EJP to research the surface morphology in sample #1. 

Sample #2 is processed by SAE and EJP combined technique; it needed to be placed in the etching slurry firstly, the etched depths in SAE processing are 20 nm, 40 nm, 60 nm, 120 nm. The surface in SAE will be analyzed. Then, sample #2 was polished by EJP technique, the removal depths are 20 nm, 40 nm, 60 nm, 80 nm, and 100 nm, and the morphology is measured. SAE parameters are listed in Table 2. EJP parameters are listed in Table 3 and kept throughout the processing.

Larger material removal may influence the actual profile of the surface and violates the original intention: recover the surface with minimal material removal. Therefore, the removal depths of two samples are within 120 nm. When roughness value returns to the initial state, the processing is stopped.

#### 2.2.2. Scanning Electron Microscope (SEM) 

SEM is used to measure the morphology of sample #1 and sample #2 (Model: PHENOM-ProX, Phenom-China, Shanghai, China). The Phenom ProX SEM is the ultimate all-in-one imaging and X-ray analysis system. Using an energy dispersive spectrometer (EDS), acquired from Phenom ProX SEM, sample structures can be physically examined and their elemental compositions determined. Besides point analysis, the optional Elemental Mapping and Line Scan software allows further analysis of the distribution of elements. Thus, the atomic percentage of metallic elements represented by aluminum (Al) can be obtained. 

#### 2.2.3. Atomic Force Microscope (AFM) 

AFM (Model: Bruker Dimension Icon, Bruker, Billerica, MA, USA) is used to measure the morphology of sample #1 and sample #2. The imaging mode in measurement is ScanAsyst. It is a PeakForce Tapping mode-based image optimization technique that enables every user to create the highest resolution AFM images using single-touch scanning. The analysis area was set to 10 μm × 10 μm and the measurement was repeated three times. The roughness root mean square values and the profile of surface microstructure of different areas are obtained by AFM.

#### 2.2.4. Weak Absorption

A photothermal absorptiometer [15] (model: PTB-1000, ZC Optoelectronic Technologies, LTD., Hefei, Anhui, China) was used to measure the weak absorption signals of sample #1 and sample #2. When irradiated by laser, high-absorption areas on the surface present as heat heaves, which can be analyzed to evaluate the surface quality and predict laser damage. The parameters are listed in Table 4, and the signal represents the average level in the measurement. To reduce the accidental error, the measurement of weak absorption is repeated for five times at the same point.

## 3. Results

### 3.1. SEM and EDS Analysis

**Figure 3:**
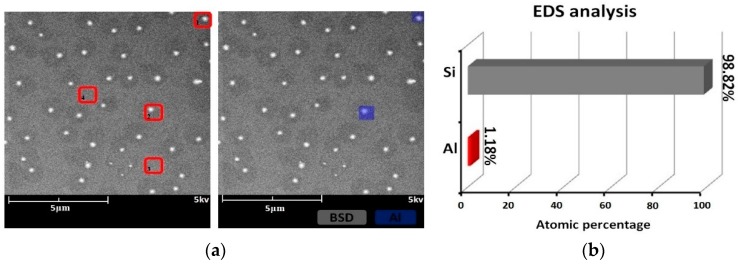
Sample #1 after IBE processing: (**a**) morphology result and (**b**) EDS result.

**Figure 4:**
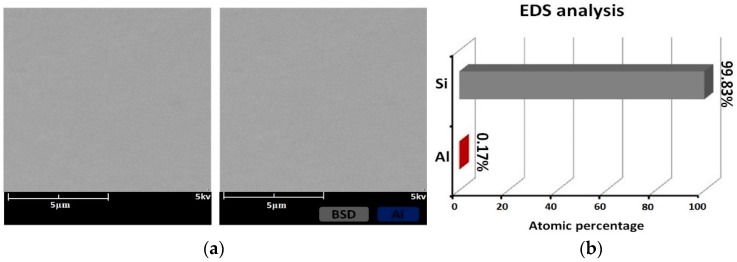
Sample #1 etched 100 nm depth in EJP processing: (**a**) morphology result and (**b**) EDS result.

**Figure 5:**
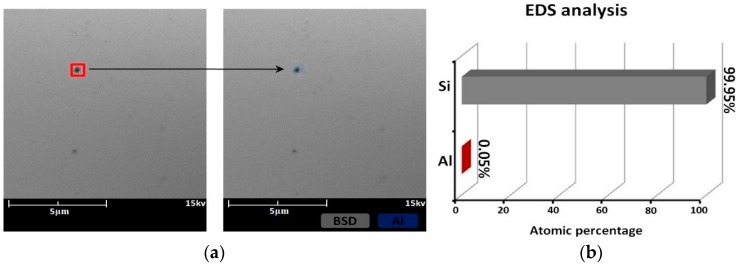
Sample #2 etched at 60 nm depth in SAE processing: (**a**) morphology result and (**b**) EDS result.

The surface morphology and EDS results in the scanning electron micrographs of sample #1 after IBE processing are shown in Figure 3. Follow IBE, a 100-nm depth is removed by EJP. The SEM results after EJP are shown in Figure 4. The results of etched 60 nm depth in sample #2 in SAE processing are shown in Figure 5. The atomic percentage of Al is marked in part (b) for Figure 3, Figure 4 and Figure 5.

In SEM, the surface of the specimen is scanned by electron beam and the morphology information is collected. For electric conductors, details of surface can be presented. By contrast, morphology information of semiconductors, including single crystal silicon, cannot be obtained entirely because of the relatively lower conductivity. Figure 3a shows that bulges exist on the component surface. During IBE processing, metallic materials can mix with silicon and then generate bulges owing to the action of the high-energy ion beam. The diameter of bulge is ~200 nm. The EDS results show that Al exists in some areas (scanning area: 1–2) and its atomic percentage is 1.18% (Figure 3b), which is different from the area where there is no bulge (scanning area: 3–4). 

In Figure 4b, it can be seen that atomic percentage of Al drops to 0.17%. According to the mechanism of EJP, any metallic contaminant should have been removed entirely, which contradicts the results of Figure 4b. That means that it exists beneath the surface.

The morphology results of sample 2# etched at 60 nm depth are shown in Figure 5a. It can be seen that bulges are removed effectively and two pits appear in SAE processing. Measured in red is the matrix zone by EDS; Al still remains embedded below the surface. Compared with Figure 3b, the atomic percentage of Al drops to 0.05% (Figure 5b), and falls by ~95%. Our results show that SAE technique has a good effect on removing metallic contaminant. After 120 nm depth etching, the atomic percentage of Al drops to 0.01%.

### 3.2. Morphology Evolution

**Figure 6:**
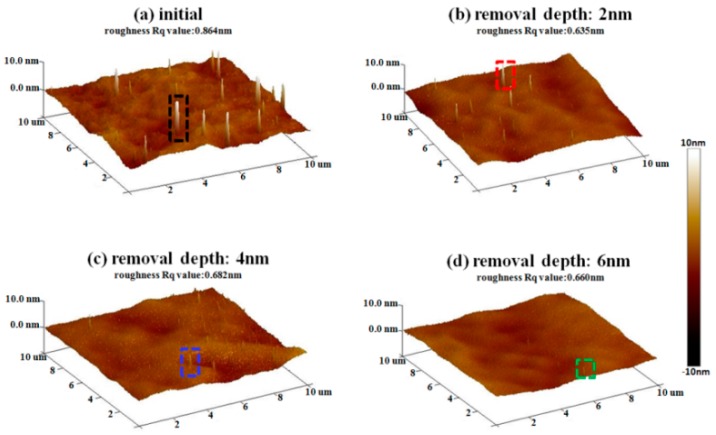
Bulges morphology evolution of sample #1 in EJP preprocessing.

**Figure 7:**
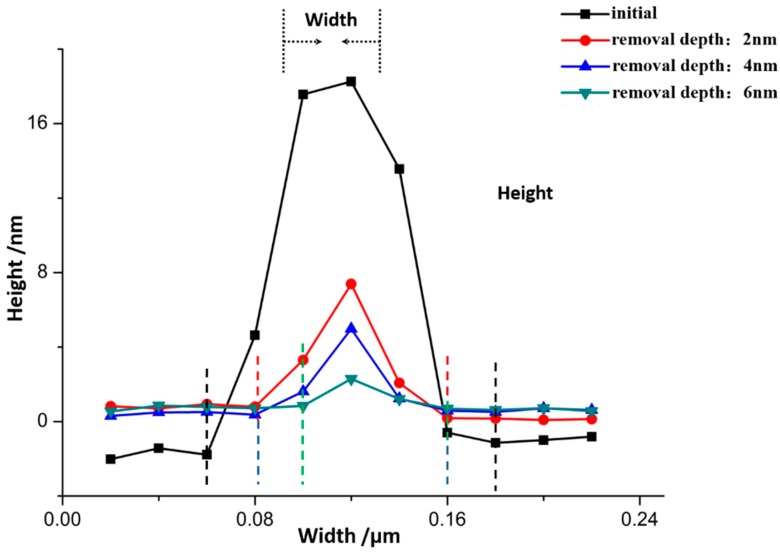
Size evolution of bulges in EJP preprocessing.

**Figure 8:**
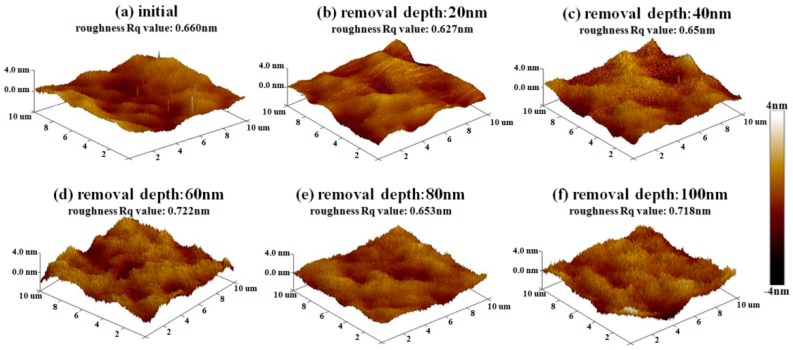
Morphology evolution of sample #1 in EJP postprocessing.

**Figure 9:**
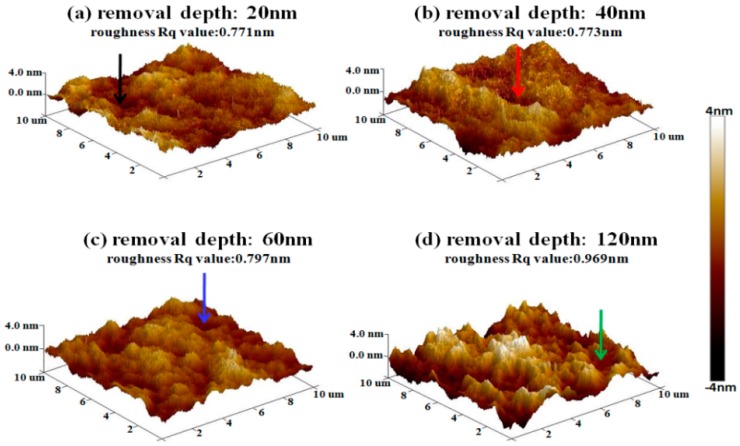
Morphology evolution of sample #2 in SAE preprocessing.

**Figure 10:**
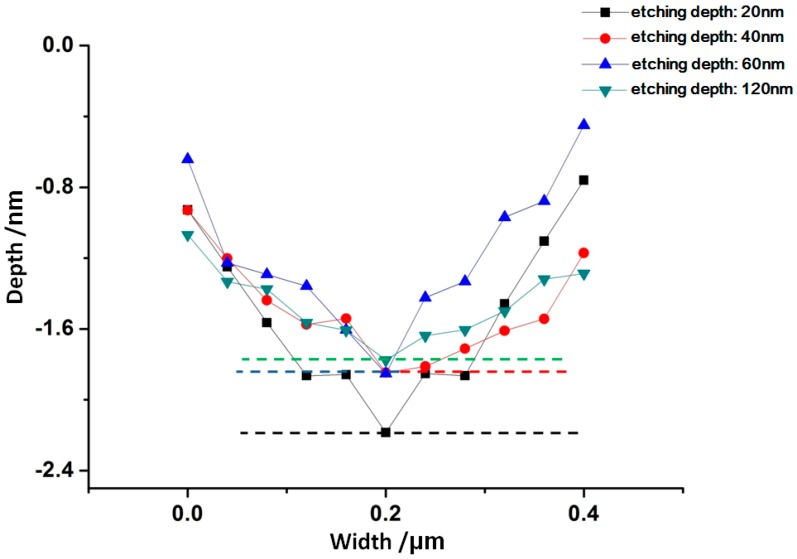
Size evolution of pits in SAE preprocessing.

**Figure 11:**
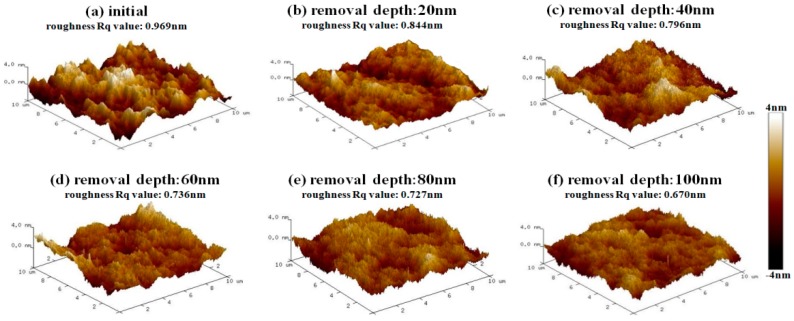
Morphology evolution of sample #2 after EJP processing.

After IBE processing, sample #1 was measured by AFM. The morphology evolution results of bulges in the early stage of EJP processing are shown in Figure 6. It must be noted that the results of Figure 7 represent morphology information of bulges that has been marked by dotted boxes in Figure 6. The evolution results of surface in EJP processing are shown in Figure 8. In Figure 9, Figure 10 and Figure 11, the results of sample #2, processed by the SAE and EJP combined technique, are described in detail.

From Figure 6a, it can be seen that bulges generates in the initial analysis area because of the action of the ion beam. The diameter of bulges is approximately 200 nm and the height is 15–20 nm. The morphology and density of bulges are similar to the results in Figure 3a. 

In Figure 7, the size evolution of bulges marked in Figure 6 is analyzed. With the increase of removal depth, the height of bulges decreases from 0.018 μm to 0.002 μm, while width decreases from 0.276 μm to 0.123 μm, the aspect ratio increases from 15.3 to 61.5. For roughness, the initial value of surface is 0.864 nm (Figure 6a). When the removal depth is 6 nm, the surface becomes smooth and its roughness value drops to 0.660 nm. Compared with Figure 6, the existence of bulges affects roughness directly. Above all, the passivation of bulges will affect the roughness value in EJP preprocessing.

Then, the results of etching at 20 nm, 40 nm, 60 nm, 80 nm, and 100 nm depths, followed by EJP surface morphology research in Sample #1, are shown in Figure 8. It can be seen that roughness Rq value fluctuates and its evolution cannot be distinguished obviously in EJP postprocessing. When removal depth comes to 100 nm, roughness rises to 0.718 nm, which indicates that surface quality of sample #1 becomes worse after EJP postprocessing.

In SAE preprocessing, some pits appear on the surface under the arrows in Figure 9. Figure 10 shows the size evolution of pits in SAE preprocessing. The depths of pits are all ~2 nm. Meanwhile, the roughness rises with the increase of removal depth. When the depth comes to 60 nm, the roughness Rq value rises to 0.797 nm. When etching continues to 120 nm, roughness continues to rise (0.969 nm). From the results, it can be seen that the surface quality of sample #2 becomes worse in SAE preprocessing, which may relate to the existence of metallic contaminant or the properties of single crystal silicon.

After SAE preprocessing, sample #2 is processed by the EJP technique, and the results are shown in Figure 11. In Figure 11c, roughness drops rapidly (0.796 nm) when the removal depth is 40 nm. When the removal depth increases to 100 nm, the roughness Rq value drops to 0.67 nm (Figure 11f), which is similar to the initial stage shown in Section 2.1. The results prove that EJP processing has an effect on reducing the roughness and smoothing the surface.

### 3.3. Weak Absorption Evolution Analysis

**Figure 12:**
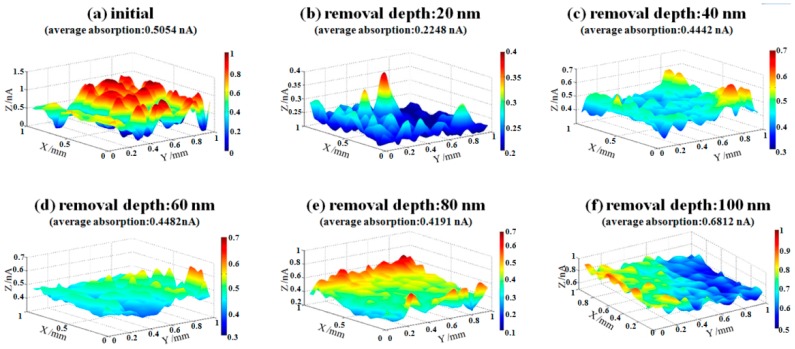
Weak absorption evolution of sample #1 in EJP processing.

**Figure 13:**
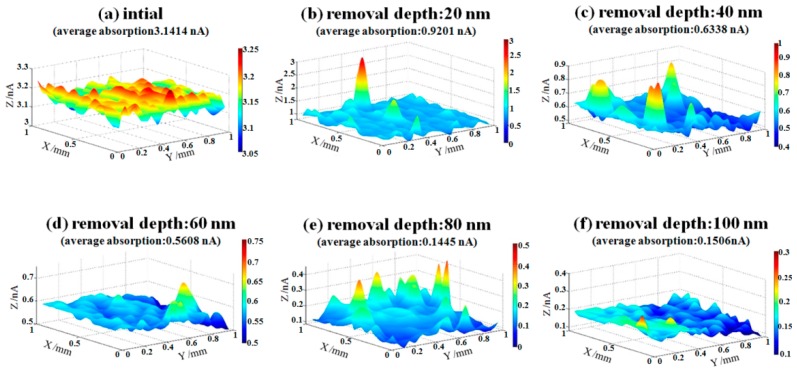
Weak absorption evolution of sample #2 in EJP processing.

In order to better evaluate the effects of EJP, a photothermal absorptiometer was used to measure the weak absorption. The value of weak absorption signal can predict the possibility of laser damage. Generally, the component which has relatively higher value of signal may be destroyed when irradiated by laser. The surface weak absorption evolution results of sample #1 and sample #2 during EJP are shown in Figure 12 and Figure 13, respectively. 

The initial weak absorption signal of sample 1# is 0.5054 nA (Figure 12a). After removing 20 nm depth, the signal drops to 0.2248 nA. When removal depth continues to increase to 80 nm in EJP processing, the signal rises to 0.4191 nA and maintains at a certain level (Figure 12c–e). When the removal depth comes to 100 nm, the signal rises to 0.6812 nA. It can be seen that weak absorption signal of sample #1 fluctuates during EJP processing, which relates to the surface conditions. 

In Figure 13, the initial weak absorption signal of sample #2 is 3.1414 nA after SAE processing (Figure 13a), and signal drops to 0.1506 nA when removal depth comes to 100 nm (Figure 13e). In the EJP processing of sample #2, the weak absorption signal continues to drop with the increase of removal depth, which means that EJP processing has a good effect on surface quality. It should be noted that the final signal may continue to drop if the removal depth continues to increase. It can be seen that bumps appear in Figure 13, which may relate to the existence of dust or other contaminants in the environment. 

## 4. Discussion

As a high-accuracy technique, IBE is used in postprocessing of optics. It uses a sputtering effect to remove material, and will not introduce damage to the surface due to the noncontact mode. Still, the technique has a few imperceptible shortcomings. Even when precautions are undertaken, metallic materials can still appear bright to optics due to the application of diaphragm or other metal components. The influence of the ion source used cannot be ignored in the introduction of contaminant either. 

Different from other postprocessing techniques, metallic materials mix with optical materials under the action of the ion beam [8]. The difference of sputtering yield makes them difficult to be removed, which may deteriorate the contamination. It is proven that the sputtering yield relates to material properties and other factors such as atomic number. Element with large atomic number has relatively higher sputtering yield, which makes sputtering of silicon prior to aluminum. Because of the mixing of metallic materials, optical surface becomes unstable and generates a structure called perturbative layer [8,9]. 

As shown in Figure 5b, aluminum still remains on the surface after 60 nm etching, although its content drops significantly compared with initial level (Figure 3b). In Figure 8, the roughness of sample #1 remains at ~0.65 nm, which differs from the tendency shown in Figure 11. It must be noted that aluminum still could be measured after EJP processing though its content remains at low levels (Figure 4b). 

**Figure 14:**
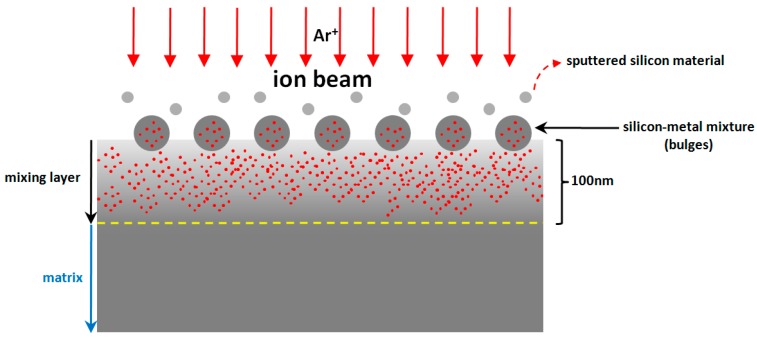
Two-phase model.

From above, metallic elements not only exist on the near surface but also beneath the surface. To explain the phenomenon better, a two-phase model is established and shown in Figure 14.

In this model, metallic elements are considered to distribute evenly beneath the surface, called the mixing layer, which is similar to the perturbative layer. Based on Section 3, the depth of mixing layer is ~100 nm, which is influenced by IBE parameters. Attributed to the long-term effect of high-energy ion beam, metallic elements mix with silicon and change the properties of materials in local areas. Because of its different atomic number aluminum sputters less than silicon in IBE processing, which promotes the generation of silicon–metal mixture on the surface. It can be seen that materials removal is faced with uncertainties due to the existence of mixing layer. Experiments in Section 3 show the influence of mixing layer and verify this model (Figure 14).

For sample #1, bulges change greatly in size during the preprocessing (Figure 6), which differs from the phenomena in IBE processing [26]. With further processing in EJP, bulges disappear with the increase of materials removal. Still, the surface roughness and weak absorption signal fluctuate (Figure 8 and Figure 12); also aluminum can be measured in the meantime. Here, the two-phase model will be applied to explain the phenomena. With the increase of removal depth in EJP processing, the deeper mixing layer is exposed and metallic elements, including aluminum, appear on the surface. Other elements decrease under the mechanical action while aluminum may react with the polishing slurry because of the alkaline environment, which would destroy the micromorphology of the surface. The negative effect of the chemical reaction has an influence on the reduction of roughness. In this period, not all metallic elements are removed entirely. A few of them still remain on the surface and can be measured by EDS (Figure 4b); their existence causes extra laser absorption, which affects the weak absorption signal (Figure 12). The reason why aluminum can be measured at the early stage of SAE processing also relates to mixing layer (Figure 5b). When mixing layer is removed entirely, the atomic percentage of aluminum drops to 0.01% that can be ignored, so as other metallic elements.

**Figure 15:**
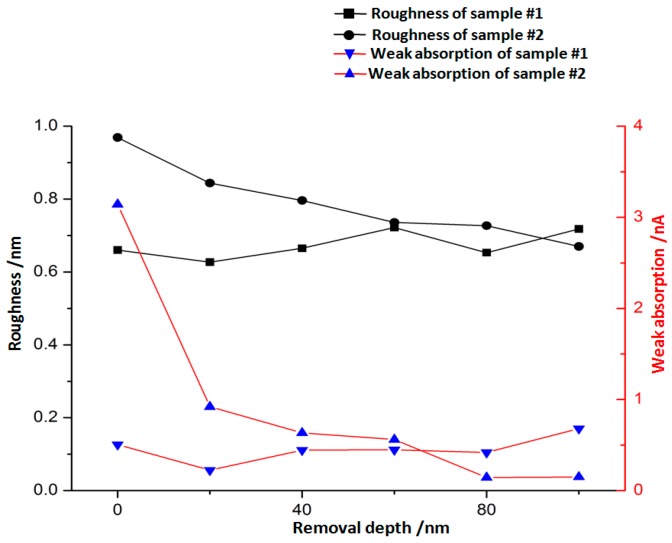
Surface revolution in EJP processing.

In Figure 15, it can be seen that the final roughness Rq value of two samples is ~0.7 nm. That means roughness reduces to the limit under the parameters listed in Table 3 when removal depth comes to 100 nm. For sample #2, EJP processing is carried out in matrix and the roughness is on a declining trend. For sample #1, the trend of roughness relates to the existence of mixing layer. 

For weak absorption, it relates to many factors. In this work, the metallic contaminant needs to be considered. It can be seen that the initial surface of sample #1 and sample #2 after removing materials to 100 nm have similar roughness values (Figure 8a and Figure 11f), but the weak absorption signal of sample #1 is three times stronger than sample #2. It also can be seen that the roughness of sample #1 (Figure 8d) is almost the same as that of sample #2 (Figure 11e), while weak absorption of sample #1 is still higher than sample #2. For sample #2, the metallic contaminant represented by aluminum is removed almost completely after SAE processing. From the above, it is proven that the difference of weak absorption can be attributed to the existence of metallic contaminant. In Figure 15, it should be noticed that the trend of roughness is consistent with that of weak absorption. However, there is no linear relationship between them. Researches have already proved that weak absorption is driven by medium-and-high frequency errors [27,28,29]. In other words, weak absorption signal is proportional to roughness value. and the effect of roughness should be considered as well. 

In this work, a weak absorption signal in the near-infrared band is measured. According to diffraction law and other researches, the weak absorption of single crystal silicon in the near-infrared band (1064 nm) coincides with that in mid-infrared band (3.8 μm), so we can analyze the damage characteristics in mid-infrared band through the results in near-infrared band [7], which may reduce the damage possibility of the components in detection process. The single crystal silicon polished by IBE technique need to be processed by other postprocessing techniques in order to reduce the negative effect of metallic contaminant on or beneath the surface. In this paper, a SAE and EJP combined technique is proposed for polishing single crystal silicon.

## 5. Conclusions

Metallic contaminant is a kind of surface defects introduced in IBE or other postprocessing, which influences the performance of single crystal silicon mirror in infrared laser system. In this work, SEM and AFM are used to characterize the metallic contaminant represented by aluminum. The results show that metallic elements exist on or beneath the surface and generate bulges under the action of high-energy ion beam. Then, EJP or the SAE and EJP combined technique is used to process the component. The roughness and weak absorption of the component were measured during the postprocessing. It can be seen that the roughness Rq value drops to 0.67 nm, while the weak absorption signal falls by 95% when processed by the SAE and EJP combined technique. To explain the phenomena in Section 3, a two-phase model was established and the principle of surface evolution was elaborated on in detail. Owing to the action of ion beam, metallic contaminant mixes with silicon and a mixing layer (~100 nm) generates, which make the surface quality fluctuate. After removing it by SAE technique, the roughness and weak absorption signal decrease linearly with the increase of removal depth. This work has elaborated the surface evolution during EJP or SAE and EJP combined technique in detail. 

Research results suggest that the SAE and EJP combined technique can be adopted in the postprocessing of single crystal silicon to improve its performance in infrared system. This preliminary work raises a series of interesting scientific questions: (a) What is the mechanism on laser damage resistance property during EJP techniques? (b) What are the key parameters in EJP techniques on polishing single crystal silicon? (c) Could the combined technique be applied on other optical materials? It is expected that further work along these lines will result in a better understanding of EJP techniques.

## Figures and Tables

**Table 1:** Parameters of IBE.

Item	Level
Vacuum chamber pressure	5 × 10^−3^ Pa or lower
Gas	Ar
Voltage	900 eV
Incident angle	0°
Spot size	Φ 15 mm
Removal rate(mm^3^/min)	11.0 × 10^−3^
Processing time	20 min

**Table 2:** Parameters of static alkaline etching (SAE).

Item	Level
Potassium hydroxide	Mass fraction: 33%
Isopropanol	Volume: 15 mL
Temperature	19 °C
Etching velocity	1 nm/min

**Table 3:** Parameters of elastic jet polishing (EJP).

Item	Level
Nozzle type	Single hole (Radius: 1.414 mm)
Polishing medium	SiO_2_ (Particle diameter: 100 nm)
PH	9~10
Pressure	0.3 MPa
Removal efficiency	1.089 × 10^−5^ mm^3^/min

**Table 4:** Parameters of weak absorption.

Item	Level
Laser wavelength	1064 nm
Power	1.0371 W
Scan size per test	1 mm × 1 mm
Pixel size	50 μm × 50 μm
Test mode	Reflect
